# Editorial on Special Issue “State-of-Art in mRNA Therapeutics and Gene Delivery”

**DOI:** 10.3390/pharmaceutics16121590

**Published:** 2024-12-13

**Authors:** Liliana S. Mendonça

**Affiliations:** 1 Center for Neuroscience and Cell Biology, University of Coimbra, 3004-504 Coimbra, Portugal; liliana.mendonca@cnc.uc.pt; 2 Center for Innovative Biomedicine and Biotechnology, University of Coimbra, 3004-504 Coimbra, Portugal

RNA therapeutics are a class of medicines based on the insertion of a specific genetic message (mRNA) into the cells and the silencing or gene editing of a specific mRNA. There are different molecules available to trigger these mechanisms, such as mRNAs, small activating RNAs (saRNAs), small interfering RNAs (siRNAs), short hairpin RNAs (shRNAs), and microRNAs (miRNAs). These molecules inherently undergo fast degradation and low cell delivery efficiency, which can be overcome with gene delivery vectors, such as viral vectors (e.g., lentiviral and adeno-associated viral particles) and non-viral vectors (e.g., lipid- and polymer-based nanoparticles) to protect and promote their cell delivery. Numerous mRNA therapeutics have been tested for immunotherapy, vaccines, and genome engineering aiming at treating and preventing various human diseases.

In this line, the Special Issue, “State-of-Art in mRNA Therapeutics and Gene Delivery”, was launched to provide an overview of the latest advances, challenges, and strengths of mRNA-based therapeutics, namely the available therapeutic molecules and delivery vectors, their modes of action, and the nonclinical and clinical development.

Six papers describing original research data were accepted and published in this Special Issue.

Double-stranded RNA (dsRNA) is an immunogenic by-product of the in vitro-transcribed (IVT) mRNA and can trigger unwanted immune responses to these IVT-mRNA medicines. Silas and colleagues, in their paper “Development of Biolayer Interferometry (BLI)-Based Double-Stranded RNA Detection Method with Application in mRNA-Based Therapeutics and Vaccines”, reported the development of a rapid, sensitive, and easy-to-implement biolayer interferometry (BLI) dsRNA detection assay using Flock House Virus (FHV) B2 protein. This assay allowed the detection of dsRNA with different uridine modifications with similar sensitivity as for dsRNA without modification and short and long dsRNA, as well as hairpin-structured dsRNA. Thus, the developed BLI method enabled the rapid and quantitative monitoring of dsRNA, regardless of nucleoside modification, in IVT mRNA products and can be easily implemented in the quality control of mRNA product manufacturing [1].

Pancreatic ductal adenocarcinoma (PDAC) is an aggressive disease characterized by multi-drug resistance, including the first-line chemotherapeutic agent gemcitabine. miR-198 has been reported to act as a tumor suppressor in PDAC, namely by targeting Valosin-containing protein (VCP). Marin-Muller et al., in their paper “Nanoparticle-Mediated Therapy with miR-198 Sensitizes Pancreatic Cancer to Gemcitabine Treatment through Downregulation of VCP-Mediated Autophagy”, reported the development and characterization of nanoparticles composed of polylactic-co-glycolic acid (LGA) and polyethyleneimine (PEI) polymers for miR-198 delivery into PDAC. The upregulation of miR-198 expression inhibited the autophagosome maturation via VCP downregulation, sensitizing PDAC cells to gemcitabine. The in vivo miR-198 delivery sensitized PDAC cells to gemcitabine, promoting reduction in tumor burden and metastatic spreading in PDAC mouse models. These data demonstrated the therapeutic effect of miR-198 in PDAC using LGA-PEI-based nanoparticles as a delivery system [2].

Sustained proteinuria causes progressive tubulointerstitial fibrosis in kidneys, mainly through the activation of proximal tubular epithelial cells (PTECs) in which the proteoglycan syndecan-1 functions as a docking platform for properdin-mediated alternative complement activation. The paper “Crotamine/siRNA Nanocomplexes for Functional Downregulation of Syndecan-1 in Renal Proximal Tubular Epithelial Cells” by Campeiro et al. described the development of the nanocomplexes composed of the cell-penetrating peptide crotamine complexed with siRNA targeting syndecan-1. Data showed that the crotamine/siRNA nanocomplexes downregulated syndecan-1 expression in PTECs in vitro in physiological and nephrotoxic conditions, reducing properdin binding to syndecan-1 and inhibiting the deposition of complement factor C3, thereby preventing complement activation. The crotamine/siRNA nanocomplexes also targeted PTECs in healthy mice, opening new paths for possible therapeutic interventions in tubulopathies [3].

Platelets can be directly activated by antisense oligonucleotides (ASOs) through nonspecific binding to platelet collagen receptor glycoprotein VI (GPVI), leading to platelet aggregation and thrombocytopenia. Valenzuela and colleagues, in their paper “Platelet Activation by Antisense Oligonucleotides (ASOs) in the Göttingen Minipig, including an Evaluation of Glycoprotein VI (GPVI) and Platelet Factor 4 (PF4) Ontogeny”, investigated the effects of a panel of ASOs on minipig platelets and the underlying mechanisms for the platelet activation and aggregation. The authors reported that adult minipig platelets were activated and aggregated by PS- and/or 2’MOE-modified ASOs; GPVI played a role in the direct activation of Göttingen minipig platelets by ASOs; and GPVI and PF4 showed a differential pattern in their protein abundance during the postnatal development of the Göttingen minipig. Furthermore, the data on direct platelet activation and aggregation by ASOs in adult minipigs was comparable to human data [4].

The lipid components and administration route of vaccines influence the intensity and quality of the humoral immune response. The paper “DLin-MC3-Containing mRNA Lipid Nanoparticles Induce an Antibody Th2-Biased Immune Response Polarization in a Delivery Route-Dependent Manner in Mice” by Yavuz and colleagues described lipid nanoparticle (LNP)-based vaccines formulated with different ionizable lipids, D-Lin-MC3-DMA (DLin) and GenVoyTM (GV), for the production of DLin- and GV-LNP encapsulating HIV-p55Gag-encoding mRNA. Mouse immunization experiments showed that although equivalent IgG kinetic profiles of general humoral responses were observed, IgG1/IgG2a ratio analysis exhibited a Th2/Th1 balance to a Th1-biased cellular immune response when both LNPs were administrated intramuscularly. Thus, independently of the ionizable lipid used in the LNPs, a Th1-biased polarization was observed for intramuscularly immunized mice. A Th2-biased antibody immunity was also observed for the DLin-containing vaccine injected subcutaneously. Therefore, data suggest that LNPs inducing a Th2- or Th1-biased adjuvant activity could be dependent on the ionizable lipid used in the formulation and the delivery route [5].

The development and implementation of efficient therapeutic strategies to reduce postischemic brain injury and stimulate regeneration is particularly important in stroke. Strategies employing the delivery of neurotrophic factors have resulted in positive therapeutic effects on the ischemic brain. Safiullov and colleagues, in their paper “Autologous Genetically Enriched Leucoconcentrate in the Preventive and Acute Phases of Stroke Treatment in a Mini-Pig Model”, tested an autologous, genetically enriched leucoconcentrate temporally secreting recombinant vascular endothelial growth factor (VEGF), glial-cell-line-derived neurotrophic factor (GDNF), and the neural cell adhesion molecule (NCAM), infused in a mini-pig ischemic stroke model. Data demonstrated improved motor performance in behavioral tests, higher preservation of brain tissue, and positive postischemic brain remodeling in the peri-infarct area, including a smaller infarct volume and number of apoptotic cells. Moreover, no immunogenic effects of the genetically modified cells at the local and systemic levels were detected. Therefore, autologous leucocytes temporarily producing therapeutic molecules may be successfully employed to treat pathological processes in the CNS such as stroke [6].
